# Optimized murine HFpEF models for translational preclinical studies

**DOI:** 10.1093/eschf/xvag072

**Published:** 2026-03-11

**Authors:** Bailey McIntosh, Ali Ali Mohamed Elbassioni, Anmar Raheem, Eilidh A MacDonald, Stuart A Nicklin, Yen Chin Koay, Ewan R Cameron, Christopher M Loughrey, John F O’Sullivan

**Affiliations:** School of Life and Environmental Sciences, Faculty of Science, The University of Sydney, Camperdown, New South Wales 2050, Australia; Cardiometabolic Medicine, Charles Perkins Centre, The University of Sydney, Camperdown, New South Wales 2050, Australia; Charles Perkins Centre, The University of Sydney, Camperdown, New South Wales 2050, Australia; The Baird Institute for Applied Heart and Lung Surgical Research, 100 Carillon Ave, Newtown, New South Wales 2042, Australia; School of Cardiovascular and Metabolic Health, Glasgow Cardiovascular Research Centre, University of Glasgow, 126 University Place, Glasgow G12 8TA, UK; School of Cardiovascular and Metabolic Health, Glasgow Cardiovascular Research Centre, University of Glasgow, 126 University Place, Glasgow G12 8TA, UK; School of Cardiovascular and Metabolic Health, Glasgow Cardiovascular Research Centre, University of Glasgow, 126 University Place, Glasgow G12 8TA, UK; School of Cardiovascular and Metabolic Health, Glasgow Cardiovascular Research Centre, University of Glasgow, 126 University Place, Glasgow G12 8TA, UK; Cardiometabolic Medicine, Charles Perkins Centre, The University of Sydney, Camperdown, New South Wales 2050, Australia; Charles Perkins Centre, The University of Sydney, Camperdown, New South Wales 2050, Australia; School of Medical Sciences, Faculty of Medicine and Health, The University of Sydney, Camperdown, New South Wales 2050, Australia; School of Biodiversity One Health and Veterinary Medicine, College of Medical Veterinary & Life Sciences, University of Glasgow, 464 Bearsden Road, Glasgow G61 1QH, UK; School of Cardiovascular and Metabolic Health, Glasgow Cardiovascular Research Centre, University of Glasgow, 126 University Place, Glasgow G12 8TA, UK; School of Biodiversity One Health and Veterinary Medicine, College of Medical Veterinary & Life Sciences, University of Glasgow, 464 Bearsden Road, Glasgow G61 1QH, UK; Cardiometabolic Medicine, Charles Perkins Centre, The University of Sydney, Camperdown, New South Wales 2050, Australia; Charles Perkins Centre, The University of Sydney, Camperdown, New South Wales 2050, Australia; The Baird Institute for Applied Heart and Lung Surgical Research, 100 Carillon Ave, Newtown, New South Wales 2042, Australia; School of Medical Sciences, Faculty of Medicine and Health, The University of Sydney, Camperdown, New South Wales 2050, Australia; Department of Cardiology, Royal Prince Alfred Hospital, 50 Missenden Road, Camperdown, New South Wales 2050, Australia

**Keywords:** Heart failure, HFpEF, Preclinical model, Hypertension, Obesity

## Abstract

**Introduction:**

The most clinically representative murine models of heart failure with preserved ejection fraction (HFpEF) include a ‘2-hit’ model combining nitrosative stress with metabolic perturbation and a ‘3-hit’ model that also includes ageing. Both models have important limitations with regard to substrain and sex.

**Methods:**

The 2-hit model protocol was modified to reproduce HFpEF in both C57BL/6N and 6J mice by increasing L-NAME doses (0.5 g/L to 1.75 g/L) and protocol lengths (7 weeks to 13 weeks). For the 3-hit model, in addition to deoxycorticosterone pivalate (DOCP), we added 1% NaCl drinking water to enhance and prolong the effect of DOCP (‘4-hit’). To maintain the phenotype, a second bolus of DOCP was administered after 8 weeks.

**Results:**

HFpEF was successfully induced in C57BL/6J mice when exposed to a 13-week 2-hit L-NAME protocol with gradually increasing dosage from 1.0 to 1.75 g/L. For the 4-hit mice, a clear HFpEF phenotype was observed in C57BL/6N and 6J mice in both male and females, and maintained for up to 12 weeks.

**Conclusion:**

These modifications ensure the 2-hit model is induced in J substrain of C57BL/6 mice. The 4-hit model prevents aldosterone escape and enhances reproducibility across sexes and substrains.

## Graphical Abstract

**Graphical Abstract:**
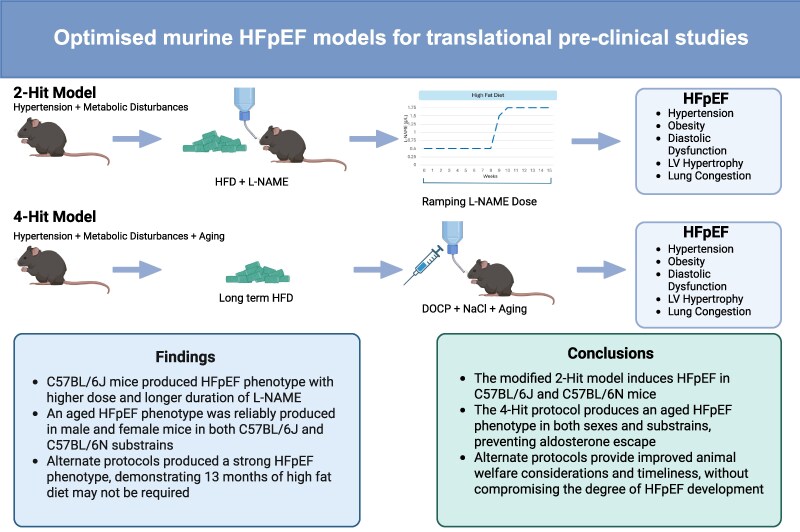
Schematic overviews of the modified 2-hit and 4-hit murine models of heart failure with preserved ejection fraction (HFpEF). Modifications to commonly used murine models of HFpEF improve phenotype reproducibility across C57BL/6J and C57BL/6N substrains.

## Introduction

Heart failure (HF) is a clinical syndrome where the heart cannot pump blood at a rate commensurate with the body’s needs (HF with reduced ejection fraction [HFrEF]) or only at the cost of increased filling pressures (HF with preserved ejection fraction [HFpEF]).^1^ HFpEF accounts for at least 50% of HF cases,^1,2^ presenting with clinical symptoms of HF such as exercise intolerance and fluid overload, frequently with associated atrial fibrillation, obesity, and metabolic syndrome.^3^

While mice have proven to be effective models for human disease, replicating complex conditions such as HFpEF remains challenging. A commonly used murine model is the ‘2-hit’ model developed by Schiattarella *et al*.,^4^ which combines a high-fat diet (HFD) to perturb metabolism with the nitric oxide (NO) synthase inhibitor L-NAME to induce hypertension and nitrosative stress. Another, ‘3-hit’, model adds ageing to hypertensive and metabolic stress^5^ and has been described as amongst the most clinically representative models.^6,7^ In this approach, 3-month-old mice are fed an HFD for 13 months to induce metabolic stress, and in the final month, deoxycorticosterone pivalate (DOCP) is administered intraperitoneally to induce hypertension and systemic inflammation.

Although both models have accelerated mechanistic research in HFpEF, several challenges and limitations remain. The 2-hit model has been successfully used in numerous studies to recapitulate key HFpEF features.^4,8,9^ However, reproducibility can vary depending on several factors including mouse substrain and sex.^10,11^ The 2-hit model is less reliable in female C57BL/6N mice.^10^ However, this protocol reproducibly produced the full spectrum of HFpEF in female C57BL/6J mice, as reported by us and others.^12–14^ Additionally, others have shown that the 2-hit model is generally more successful in N than J strains, regardless of sex.^11^ The majority of genetically modified mouse models are generated on a C57BL/6J background and so a reproducible HFpEF model in the J strain is critical for mechanistic study. With regards to the ‘3-hit’ model, there are considerable challenges regarding reproducibility and phenotypic robustness. Therefore, herein, we sought to modify the ‘2-hit’ and ‘3-hit’ protocols for improved reproducibility and clinical modelling.

## Methods

### Animal husbandry and HFpEF models

Animal experiments were performed under the approval of University of Glasgow Animal Welfare and Ethical Review Body, Glasgow, UK (project license no. P05FEIF82 and subsequently PP4465428) and the Animal Ethics Committee of The University of Sydney (Project 2023/2274), Sydney, Australia. Animals were housed in controlled environments with a 12-hour light/dark cycle and had access to food and water *ad libitum*.

For the 2-hit protocol, 10–12 week C57BL/6J male mice were purchased from Envigo, UK, while C57BL/6N male mice were obtained from Jackson Laboratory, and the colony was bred and maintained in the animal house. Animals were assigned to either a control group (standard chow diet RM1 (P) 801151, Special Diets Services) or the HFpEF group (HFD [Research Diets Inc; D12492i] and L-NAME [Sigma Aldrich, UK; N5751-25G] at concentrations ranging from 0.5 to 1.75 g/L, pH adjusted to 7.4). Mice were kept on HFD + L-NAME between 7–15 weeks to induce HFpEF.

For the 3- and 4-hit protocols, 6-week male and female mice were purchased from Animal Bioresources. At 12 weeks of age, mice were randomly assigned to either the control group (standard brown chow, Specialty Feeds Ltd SF00-100) or HFpEF group (HFD with 60% energy from lard, Specialty Feeds SF18-072) and were maintained on the diets for the period denoted on their relevant protocols (e.g. 6 months for refined protocol or 13 months for full length). After either 6 or 13 months of diet, the HFpEF groups received an intraperitoneal injection of DOCP (75 mg/kg, Zycortal Suspension). One week after DOCP injection, mice started 1% NaCl drinking water (793566, Merck) which continued to the end of the protocol. All experiments were performed in male and female mice, and animals were randomly allocated to procedure groups.

### Exercise testing

For the 2-hit model, mice underwent a 3-day acclimatization period for 10–15 min each day on the forced exercise wheel (Lafayette instruments) prior to the intolerance test. During the intolerance test, mice began with a 5-min warm-up at 3 m/min, after which the speed was increased to 4 m/min and maintained until the animals reached exercise intolerance.^15^ Exercise intolerance was defined as the point at which a mouse could not resume running within 10 s after coasting inside the wheel.

For the 4-hit model, mice were acclimatized to the treadmill (Ugo Basile) for 15 min on three consecutive days. The treadmill was set at a 20° incline and increased at a rate of 2 m/min until the animals were exhausted.^4^ Exhaustion was defined by the animal’s refusal to run after three pushes on the back with a brush. The running time and distance were recorded.

### Blood pressure

Blood pressure was measured non-invasively in conscious mice using the BIOSEBLAB instruments BP-2000 Blood Pressure Analysis System™ (for the 2-hit model), and the Kent CODA high throughput 6-channel instrument (for the 4-hit model). Mice were acclimatized to the restrainers for 10 min per day, for 3 consecutive days. For testing, the restrainer holding the animal was placed on a 37°C warming tray and covered with a blanket. After allowing 5–10 min for the animals to settle, the occlusion and volume pressure recording cuffs were placed around the tail. 20–25 readings were taken, including 10 acclimatization readings and 10–15 test readings. The measurements were repeated on at least 2 different days. Animals were not restrained or left in a heated chamber for more than 30 min. All blood pressure measurements displayed in this manuscript were acquired using a tail cuff.

### Echocardiography

Echocardiography was performed using Siemens Acuson Sequoia (for the 2-hit model) and Fujifilm VisualSonics VEVO F2 LAZR-X with a UHF57X transducer (for the 4-hit model). Anaesthesia was induced using 3% isoflurane for 3 min. Isoflurane was reduced to 1.5–2.5% and adjusted as necessary to maintain the heart rate between 400 and 500 bpm.^16^ Left ventricular ejection fraction (LVEF) and global longitudinal strain (GLS) measurements were obtained from a parasternal long axis view, and transmitral Doppler measurements and tissue Doppler measurements at the mitral annulus were obtained from an apical four chamber view. Images were stored digitally and analysed blindly using ImageJ and VisualSonics workstation. A full summary of echocardiography parameters can be found in Supplementary Table S1.

### Organ weighing

Organs were weighed using an Ohaus Pioneer PX Analytical Balance. Animals were either anaesthetized with 4% isoflurane (maintenance: 2%), and reflexes checked before thoracotomy (2-hit model), or pentobarbitol anaesthesia used (100 mg/kg of a 25 mg/ml solution, intraperitoneal injection) and reflexes checked before thoracotomy (4-hit model). The heart and lungs were excised, washed with saline, and separated. The aorta was cannulated, and the heart flushed with saline to clear blood, dried, and then weighed. Lungs were cleaned of excess tissues and weighed. Lungs were dried using a Speed-Vac Concentrator until weight stabilized after which the wet-to-dry lung weight ratios were calculated.^4^

### Statistical analysis

Statistical analysis was made using GraphPad prism version 10.4.1. Comparisons between two groups were performed depending on inbuilt normality tests, with unpaired *t*-test used for parametric data and Mann–Whitney test used for nonparametric data. *P*-value threshold was 0.05. Individual data points on graphs represent one mouse. Graphs display mean ± SEM.

## Results

**Figure 1:**
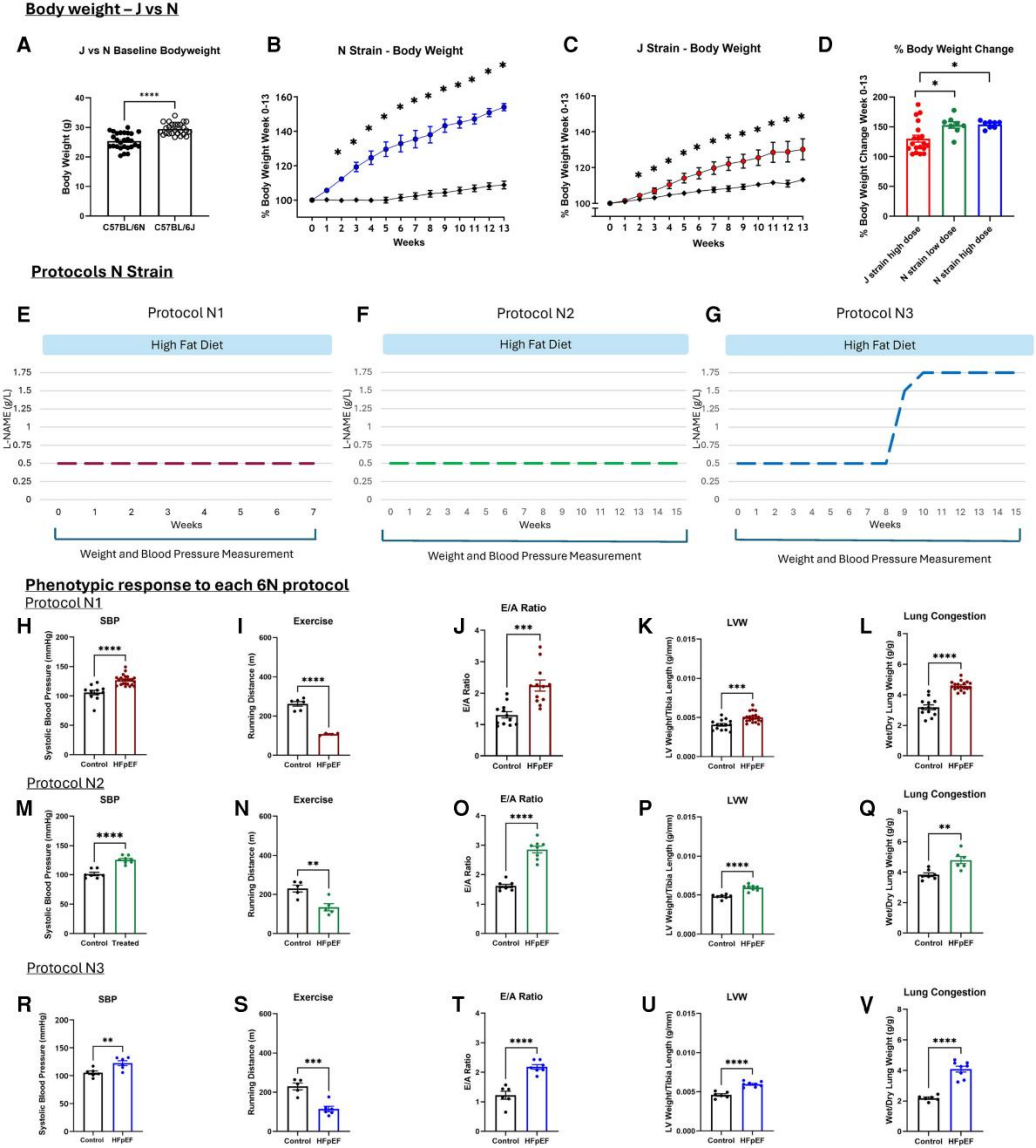
N-strain mice produce robust HFpEF phenotype in response to all protocols. (*A*) Baseline body weight of 6J and 6N mice (*n* = 27 for 6N mice, *n* = 26 for 6J mice). (*B*) Response of N-strain body weight to 13 weeks of HFD and L-NAME (*n* = 6 for control, *n* = 8 for HFpEF). (*C*) Response of J-strain body weight to 13 weeks of HFD and L-NAME (*n* = 26 for control, *n* = 25 for HFpEF). (*D*) Percentage body weight change of J- and N-strain mice after 13 weeks of HFD, and high (1–1.75 g/L ramping dose for 6J and 0.5–1.75 g/L ramping dose for 6N) or low dose (0.5 g/L) L-NAME (*n* = 19 for J-strain high dose, *n* = 8 for N-strain low dose, *n* = 8 for N-strain high dose). (*E*) Schematic of 6N protocol N1, showing 7 weeks of low dose L-NAME. (*F*) Schematic of 6N protocol N2, showing 15 weeks of low dose L-NAME. (*G*) Schematic of 6N protocol N3, showing an increasing dose of L-NAME over 15 weeks. (*H–L*) Depict measurements taken at the end of the N1 protocol. (*H*) SBP of control and HFpEF mice (*n* = 11 for control, *n* = 24 for HFpEF). (*I*) Running distance of control and HFpEF mice (*n* = 6 for control, *n* = 4 for HFpEF). (*J*) Echocardiography measurement representing peak velocity of mitral blood flow at early filling, to peak velocity of mitral blood flow at late filling (E/A ratio) (*n* = 12 for control, *n* = 12 for HFpEF). (*K*) LVW normalized to tibia length as a measurement of LV hypertrophy (*n* = 14 for control, *n* = 20 for HFpEF). (*L*) Ratio of wet to dried lung weight as a measurement of lung congestion (*n* = 13 for control, *n* = 18 for HFpEF). (M-Q) Depict measurements taken at the end of the N2 protocol. (*M*) SBP of control and HFpEF mice (*n* = 7 for control, *n* = 8 for HFpEF). (*N*) Running distance of control and HFpEF mice (*n* = 5 for control, *n* = 5 for HFpEF). (*O*) E/A ratio (*n* = 8 for control, *n* = 8 for HFpEF). (*P*) LV hypertrophy (*n* = 7 for control, *n* = 8 for HFpEF). (*Q*) Wet/dry lung ratio (*n* = 7 for control, *n* = 6 for HFpEF). (*R–V*) Depict measurements taken at the end of the N3 protocol. (*R*) SBP of control and HFpEF mice (*n* = 6 for control, *n* = 6 for HFpEF). (*S*) Running distance of control and HFpEF mice (*n* = 5 for control, *n* = 6 for HFpEF). (*T*) E/A ratio (*n* = 6 for control, *n* = 7 for HFpEF). (*U*) LV hypertrophy (*n* = 6 for control, *n* = 8 for HFpEF). (*V*) Wet/dry lung ratio (*n* = 6 for control, *n* = 8 for HFpEF). *P < .05, **P < .01, ***P < .001, ****P < .0001 by unpaired *t*-test. Graphs display ± SEM

**Figure 2:**
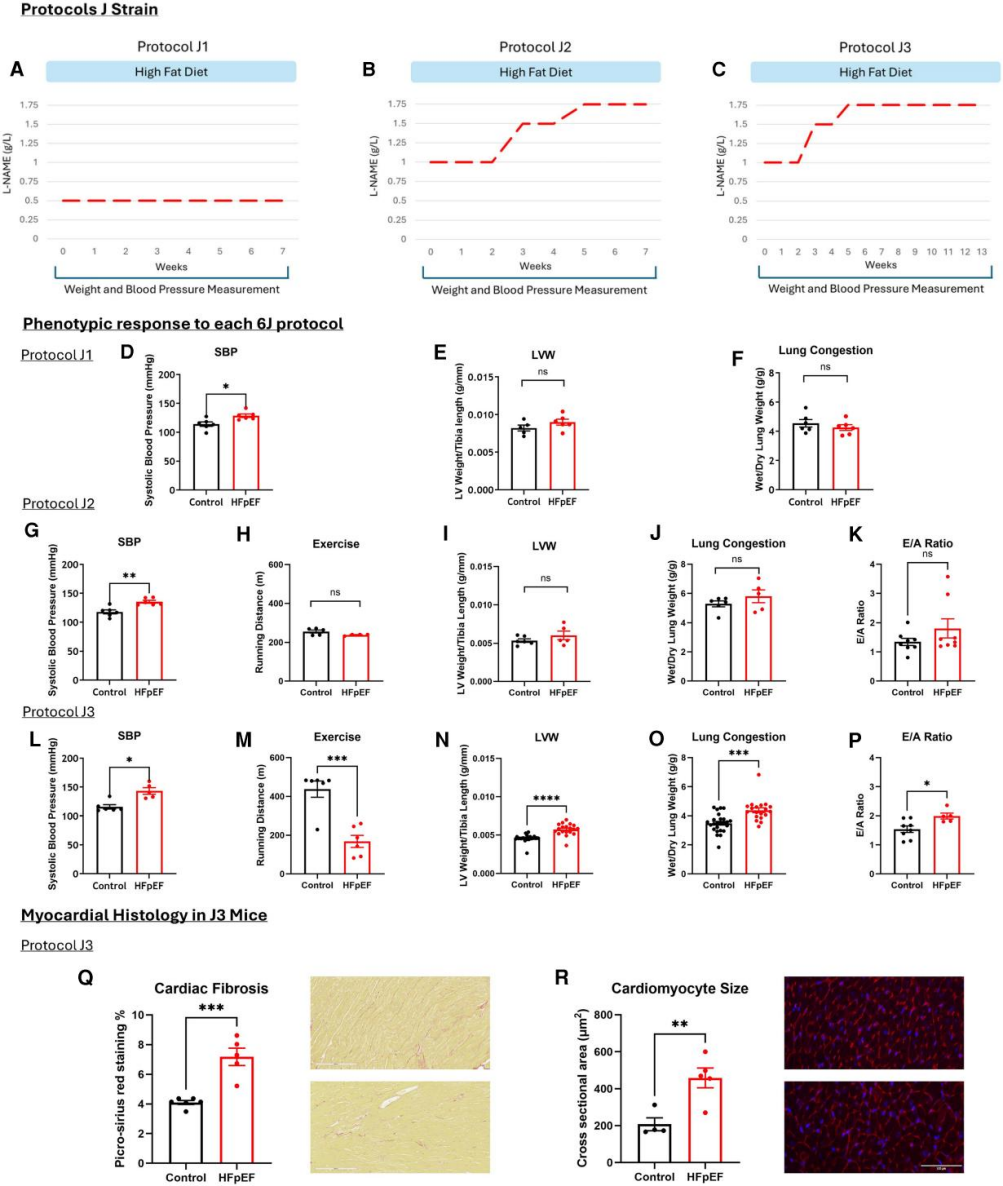
Protocol testing to produce robust HFpEF phenotype in male J strain. (*A*) Schematic of 6J protocol J1, showing 7 weeks of low dose L-NAME. (*B*) Schematic of 6J protocol J2, showing an increase of L-NAME over 7 weeks. (*C*) Schematic of 6J protocol J3, showing an increase of L-NAME which is maintained for 13 weeks. (*D*) SBP at the end of J1 protocol (*n*  *&* 6 for control, *n* = 6 for HFpEF. (*E*) LV hypertrophy at the end of J1 protocol (*n* = 5 for control, *n* = 6 for HFpEF). (*F*) Wet/dry lung ratio at the end of J1 protocol (*n* = 6 for control, *n* = 6 for HFpEF). (*G–K*) show measurements from the end of J2 protocol. (*G*) SBP at the end of J2 protocol (*n* = 6 for control, *n* = 6 for HFpEF). (*H*) Running distance of control and HFpEF mice (*n* = 5 for control, *n* = 4 for HFpEF). (*I*) LV hypertrophy (*n* = 6 for control, *n* = 5 for HFpEF). (*J*) Wet/dry lung ratio (*n* = 6 for control, *n* = 5 for HFpEF). (*K*) E/A ratio (*n* = 8 for control, *n* = 8 for HFpEF). (*L–P*) Measurements from the end of J3 protocol. (*L*) SBP at the end of J3 protocol (*n* = 6 for control, *n* = 5 for HFpEF). (*M*) Running distance of control and HFpEF mice (*n* = 6 for control, *n* = 6 for HFpEF). (*N*) LV hypertrophy (*n* = 26 for control, *n* = 19 for HFpEF). (*O*) Wet/dry lung ratio (*n* = 25 for control, *n* = 19 for HFpEF). (*P*) E/A ratio (*n* = 8 for control, *n* = 5 for HFpEF). (*Q*) Increased cardiac fibrosis in J3 mice as shown by picro-sirius red staining (*n* = 6 for control, *n* = 5 for HFpEF). (*R*) Cardiomyocyte size in protocol J3 mice (*n* = 4 for control, *n* = 5 for HFpEF). *P < .05, **P < .01, ***P < .001, ****P < .0001 by unpaired *t*-test. Graphs display ± SEM

Previously, it has been suggested that C57BL/6N mice may be more susceptible to the standard 2-hit model than C57BL/6J animals (Pepin *et al.*, 2025^11^) To better understand the comparative susceptibilities of N and J strains, we explored their responses to different treatment regimens that varied both in the dosage and the length of treatment of L-NAME (*Figures 1* and *2*). The full HFpEF phenotype was determined to have been induced if there were significant changes in E wave (early diastole)/A wave (late diastole) ratio, E/e′ (e prime tissue Doppler wave) ratio, exercise capacity, left ventricular mass, blood pressure, body weight, and lung congestion as indicated by increased wet/dry weight lung mass ratio.

### Both N and J strain respond to HFD and L-NAME

There are contradictory reports in the literature as to whether N- or J- strain mice are intrinsically heavier.^17^ Our results show that age-matched J-strain animals had a small but significantly higher baseline body weight than their N-strain counterparts (*Figure 1A*). In agreement with data reported by Nemoto *et al*.,^17^ both strains showed significant weight gain on an HFD (*Figure 1B* and *C*), with percentage weight gain being significantly higher in N-strain animals after 13 weeks of HFD (*Figure 1D*). These results contrast with others^11^ who found that mice lacking a functional NNT gene, as is the case in the J strain, did not show significant weight gain on an HFD. Within the more responsive N strain, animals on an HFD showed similar increases in weight using low-dose (0.5 g/L, protocol N2) or high-dose (0.5–1.75 g/L, protocol N3) L-NAME (*Figure 1D*).

### Higher doses of L-NAME do not augment the HFpEF phenotype in N-strain mice

We next examined whether increasing doses of L-NAME impacted the HFpEF phenotype in N-strain mice. Although the length of treatment varied, a single HFD was used throughout. We applied three protocols to the N-strain mice: HFD + 0.5 g/L L-NAME for 7 weeks (Protocol N1, *Figure 1E*), HFD + 0.5 g/L L-NAME for 15 weeks (Protocol N2, *Figure 1F*), or HFD + 0.5 g/L L-NAME for 8 weeks, followed by 1.5 g/L for 2 weeks, and then 1.75 g/L for 5 weeks (Protocol N3, *Figure 1G*).

As expected, fractional shortening did not change in these protocols (Supplementary Figures S1A and B). Although there was some weekly variation, animals treated with 0.5 g/L L-NAME showed a rise in systolic blood pressure (SBP) after 1 week of treatment and plateaued thereafter. Continued treatment up to 7 weeks (Protocol N1, *Figure 1H*) led to significantly increased blood pressure, reduction in running distance (*Figure 1I*), increase in E/A ratio (*Figure 1J*), increase in left ventricular weight (*Figure 1K*), and increase in wet/dry lung weight (*Figure 1L*). The N2 (*Figure 1M–Q*) and N3 (*Figure 1R–V*) protocols also led to significant changes in these parameters, but no more markedly than the N1 protocol. These results demonstrate that in N-strain animals, a robust HFpEF phenotype can be achieved after 7 weeks at an L-NAME dose of 0.5 g/L and that higher doses do not augment the phenotype further.

### HFpEF can be induced in J-strain mice

Producing a robust phenotype in the J strain would facilitate research using genetic mice models which are mostly based on the J strain. Given previous reports of resistance to HFpEF in the J strain (Pepin *et al*., 2025^11^) we tested different protocols, varying the length of treatment and dose of L-NAME, to determine whether a robust phenotype is possible in the male J strain. As protracted 15-week L-NAME treatment at 0.5 g/L did not further augment blood pressure, we changed to a ramped dose protocol as per below.

J-strain mice treated for 7 weeks at doses of L-NAME ranging from 0.5–1.75 g/L (Protocols J1, *Figure 2A*; J2, *Figure 2B*) all developed hypertension but failed to show the full range of signs indicative of HFpEF (*Figure 2D–K*). This contrasts with the N strain where 7 weeks of HFD and 0.5 g/L of L-NAME could achieve a comprehensive HFpEF phenotype. However, J-strain mice treated for 13 weeks with a dosage regime rising from 1.0 to 1.75 g/L (Protocol J3, *Figure 2C*) showed significant changes in SBP, E/A ratio, exercise tolerance, left ventricular mass, and lung wet/dry ratio (*Figure 2L–P*). In addition, postmortem histological examination revealed increased cardiac fibrosis and increased cardiomyocyte cross sectional area indicating cardiac hypertrophy (*Figures 2Q* and *R*). Importantly, the fractional shortening was preserved in these animals (Supplementary Figure S1C and D). These results clearly demonstrate that it is possible to induce a HFpEF phenotype in C57BL/6J mice, in which we also confirmed histologically the presence of cardiomyocyte hypertrophy and extracellular myocardial fibrosis.

### Augmenting the phenotype in 3-hit mice: the ‘4-hit’ protocol

**Figure 3:**
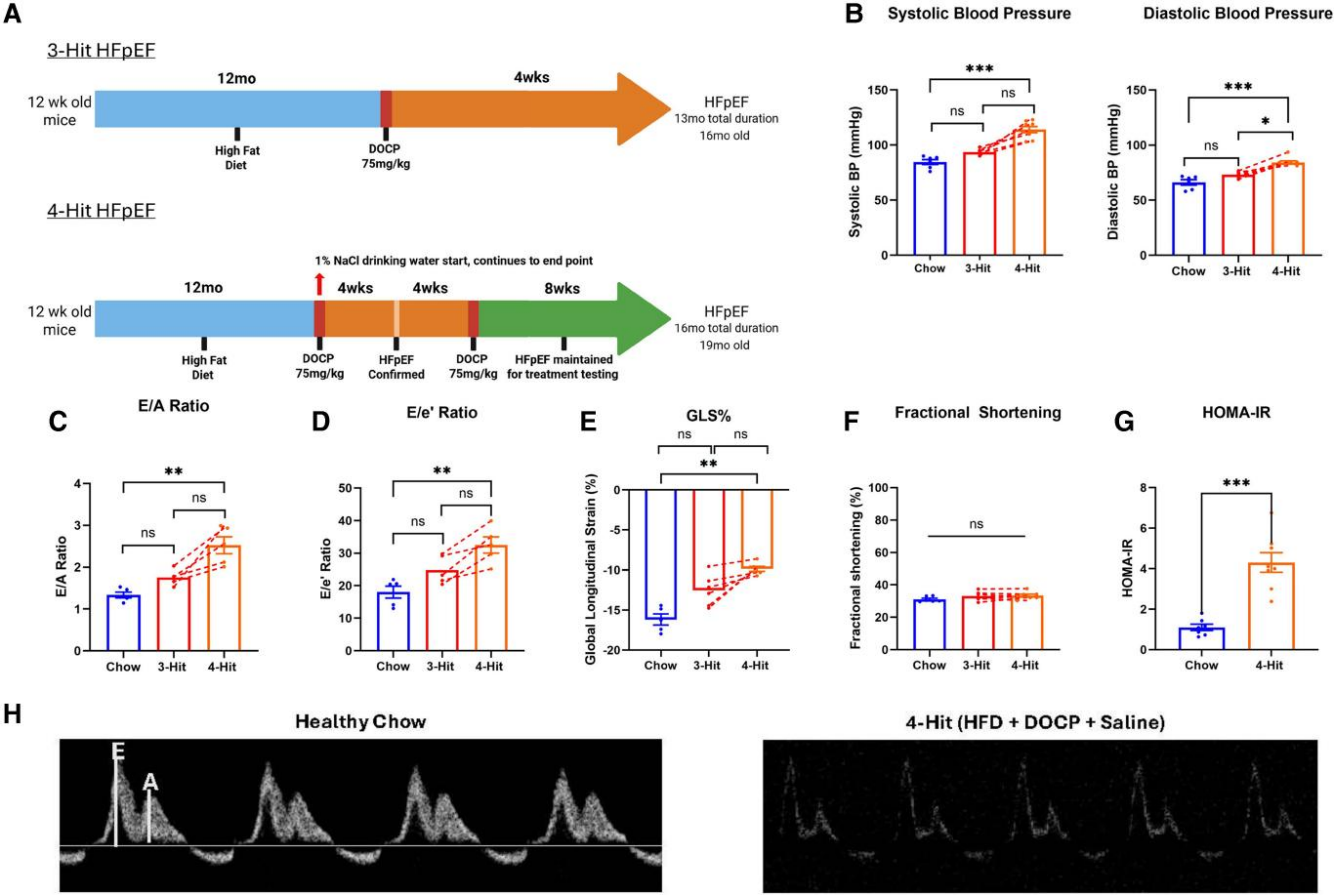
A reproducible HFpEF phenotype incorporating ageing—‘4-hit’. (*A*) Schematic depicting the traditional 3-hit approach, and our modified 4-hit approach incorporating NaCl drinking water and an additional DOCP injection. (*B*) SBP and DBP after the traditional 3-hit approach in red and following the addition of NaCl in orange. (*C*) E/A ratio before and after NaCl. (*D*) Ratio of peak velocity of mitral blood flow at early filling to peak early diastolic mitral annulus velocity (E/e′) before and after the addition of NaCl. (*E*) GLS of the left ventricle. (*F*) Preserved fractional shortening of the left ventricle. (*G*) HOMA-IR of healthy chow mice and 4-hit mice at sacrifice. (*H*) Representative pulsed wave Doppler images for chow and 4-hit groups. For all panels, male C57BL/6N mice were used. For A–G, *n* = 6 for chow, *n* = 7 for 3-hit, *n* = 7 for 4-hit. *P < .05, **P < .01, ***P < .001 by Mann–Whitney test. Graphs display ± SEM

**Figure 4:**
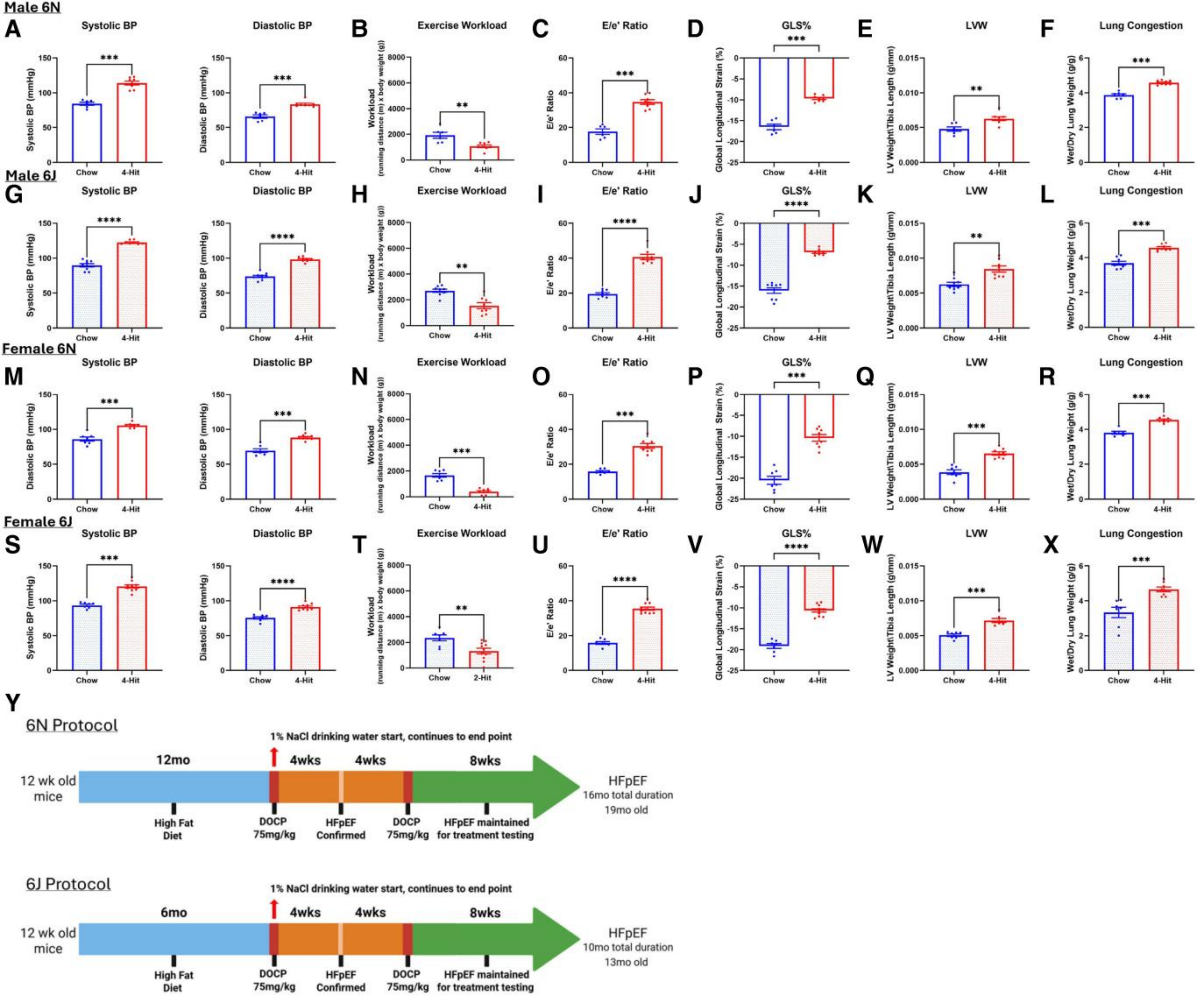
4-Hit model reproducibly produces HFpEF in male and female J and N. (*A–F*) Measurements from male 6N mice at the end of 4-hit protocol. (*A*) SBP and DBP of chow and 4-hit male 6N mice. (*B*) Exercise workload (body weight × running distance) of chow and 4-hit mice. (*C*) Ratio of peak velocity of mitral blood flow at early filling to peak early diastolic mitral annulus velocity (E/e′ ratio). (*D*) GLS of the left ventricle. (*E*) LV hypertrophy. (*F*) Wet/dry lung ratio. For A–F, *n* = 7 for chow, *n* = 8 for 4-hit. (*G–L*) Measurements from male 6J mice at the end of 4-hit protocol. (*G*) Systolic and diastolic blood pressure. (*H*) Exercise workload. (*I*) E/e′ ratio. (*J*) GLS of the left ventricle. (*K*) LV hypertrophy. (*L*) Wet/dry lung ratio. For G–L, *n* = 9 for chow, *n* = 8 for 4-hit. (*M–R*) Measurements from female 6N mice at the end of 4-hit protocol. (*M*) SBP and DBP. (*N*) Exercise workload. (*O*) E/e′ ratio. (*P*) GLS of the left ventricle. (*Q*) LV hypertrophy. (*R*) Wet/dry lung ratio. For M–R, *n* = 7 for chow, *n* = 8 for 4-hit. (*S–X*) Measurements from female 6J mice at the end of 4-hit protocol. (*S*) SBP and DBP. (*T*) Exercise workload. (*U*) E/e′ ratio. (*V*) GLS of the left ventricle. (*W*) LV hypertrophy. (*X*) Wet/dry lung ratio. For S–X, *n* = 7 for chow, *n* = 10 for 4-hit. (*Y*) Schematics of the relevant protocols applied to each strain. **P < .01, ***P < .001, ****P < .0001 by Mann*–*Whitney test. Graphs display ± SEM

The 3-hit model described by Deng *et al.*^5^ in 2021 is schematically outlined alongside our modification with NaCl (‘4-hit’) in *Figure 3A*. Using the 3-hit model, we did not see a significant elevation of SBP at 13 months in male C57BL/6N mice, whereas we did after 1% NaCl was added to drinking water (*Figure 3B*; left). We saw a similar pattern for DBP (*Figure 3B*; right), E/A ratio (*Figure 3C*), E/e′ ratio (*Figure 3D*), and GLS (*Figure 3E*). Left ventricular fractional shortening (*Figure 3F*) and ejection fraction (Supplementary Figure S1E) were preserved in both protocols, and 4-hit mice had a significantly worse homeostatic model of insulin resistance (*Figure 3G*). Due to the observed enhancement of the HFpEF phenotype with the addition of 1% NaCl drinking water, it was provided 1 week post DOCP injection in subsequent batches and referred to as 4-hit model. When monitoring blood pressure following the administration of DOCP and saline, hypertension was observed to decrease approximately 7 weeks after the initial DOCP injection despite the additional saline challenge (Supplementary Figure S1F–G). This observation confirmed that NaCl alone was not inducing the hypertensive effect. We also determined the mice could tolerate a second dose of DOCP with no adverse response, allowing the HFpEF phenotype to be sustained longer. Further, we demonstrated we could recreate the HFpEF phenotype with the 4-hit protocol in a shorter timeframe, as demonstrated in the C57BL/6J 4-hit mice in which the initial HFD loading phase was shortened by 5 months (Schematic in Supplementary Figure S2A) in male (*Figure 4G–L*) and female mice (*Figure 4S–X*). Importantly, ejection fraction and fractional shortening were maintained in all cohorts (Supplementary Figures S1H–O).

To ensure the aged C57BL/6J mice still responded to the DOCP and NaCl in the expected manner, a group of 6J male mice were aged to 15 months, injected with DOCP, and given NaCl drinking water. We observed the same increase in systolic and diastolic blood pressure (Supplementary Figure S2B), and as inducing sustained hypertension is the main limiting factor in aged HFpEF models, there are no indications our findings would not translate if the protocol was not shortened by 5 months in this strain.

We next proceeded to examine the key HFpEF parameters in the standard and modified 4-hit models (Schematic *Figure 4Y*) model across substrain and sex (*Figure 4*). SBP was significantly elevated in the 4-hit model in male 6N mice, as was DBP (*Figure 4A*), with significant reduction in exercise workload (*Figure 4B*), increase in E/e′ ratio (*Figure 4C*), significant decrease in GLS (*Figure 4D*), significant increase in LV weight (*Figure 4E*), and significant increase in wet/dry lung weight (*Figure 4F*). The same pattern was seen in male 6J (*Figure 4G–L*), female 6N (*Figure 4M–R*), and female 6J mice (*Figure 4S–X*). This thorough phenotyping confirms the model is reproducible across strains and sexes, and the refined model is still sufficiently robust to produce a complete phenotype in the ‘less susceptible’ 6J substrain.

### Phenotype comparison: 4-hit vs 2-hit

**Figure 5:**
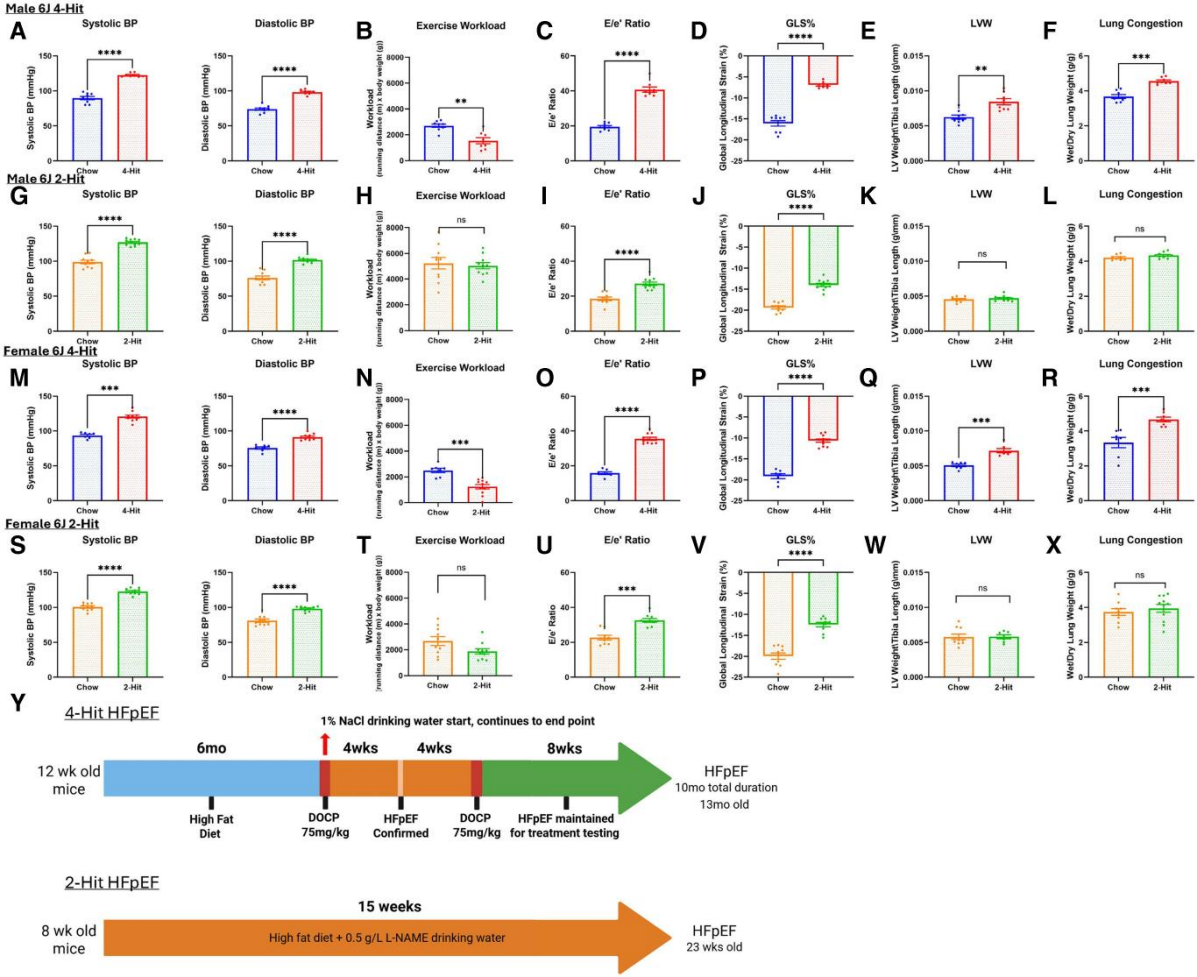
Phenotypic comparison of 4-hit (incorporating ageing) vs standard 2-hit (without ageing). 4-hit data are duplicated from *Figure 4*. (*A–F*) Measurements from 6J males at completion of 4-hit protocol. (*A*) SBP and DBP. (*B*) Exercise workload. (*C*) E/e′ ratio. (*D*) GLS of the left ventricle. *E*. LV hypertrophy. (*F*) Wet/dry lung ratio. For A–F, *n* = 7 for chow, *n* = 8 for 4-hit. (*G–L*) Measurements from 6J males at completion of the standard 2-hit protocol. (*G*) SBP and DBP. (*H*) Exercise workload. (*I*) E/e′ ratio. (*J*) GLS of the left ventricle. (*K*) LV hypertrophy. (*L*) Wet/dry lung ratio. For G–L, *n* = 10 for chow, *n* = 11 for 2-hit. (*M–R*) Measurements from 6J females at completion of 4-hit protocol. (*M*) SBP and DBP. (*N*) Exercise workload. (*O*) E/e′ ratio. (*P*) GLS of the left ventricle. (*Q*) LV hypertrophy. (*R*) Wet/dry lung ratio. For M–R, *n* = 7 for chow, *n* = 10 for 4-hit. (*S–X*) Measurements from 6J females at completion of the standard 2-hit protocol. (*S*) SBP and DBP. (*T*) Exercise workload. (*U*) E/e′ ratio. (*V*) GLS of the left ventricle. (*W*) LV hypertrophy. (*X*) Wet/dry lung ratio. (*Y*) Schematics depicting the relevant 4-hit and 2-hit protocols utilized in this figure. For S–X, *n* = 10 for chow, *n* = 10 for 2-hit. **P < .01, ***P < .001, ****P < .0001 by Mann–Whitney test. Graphs display ± SEM

We next compared the 4-hit to the traditional ‘standard’ 2-hit HFpEF phenotypic parameters in male and female J-strain mice. These 2-hit mice followed the protocol as outlined in *Figure 1F*, 15 weeks of 0.5 g/L of L-NAME,^4^ though in C57BL/6J background, not 6N (as in Schiattarella *et al*.). In both male and female 6J mice, significance was observed in blood pressures, E/e′ ratio, and GLS using the unmodified 2-hit model, but not in exercise workload, left ventricular mass, and lung congestion (*Figure 5G–L, S–X*).

However, if one considers the presence of all parameters necessary for a successful model, then the 4-hit model (*Figure 5A–F, M–R*) was superior to the standard 2-hit model in capturing the full phenotype in 6J mice. The displayed phenotypes are crucial parameters of HFpEF and were not observed in 2-hit mice using the unmodified protocol, highlighting the importance of the modifications to the model outlined here. All 4-hit protocols across sexes and strains, and the unmodified 2-hit protocol in both sexes in 6J mice, developed a higher homeostatic model assessment of insulin resistance (HOMA-IR) compared to the control groups (Supplementary Figure S2C).

## Discussion

While murine models play a very important role in the investigation of HFpEF, capturing a more complete clinical picture of the disease state with such complex pathophysiology is not without limitations. The aetiology of insidious onset cardiac disease, such as HFpEF, is complex and derives from multiple, interlinked predisposing risk factors such as age, hypertension, type II diabetes, obesity, kidney disease, COPD, anaemia, and sleep disordered breathing.^18,19^ This complex causation is paralleled by the complexity of the different pathophysiological changes associated with the clinical presentation of HFpEF including cardiac fibrosis and stiffness, metabolic changes, mitochondrial and energy dysfunction, microvascular disease, and inflammatory changes, all of which contribute to reduced ventricle compliance, suboptimal diastolic filling, and increased filling pressures.^2,20–22^

If mouse models are to be useful, the minimum requirements are that they are robust and the results are highly repeatable. Only if these conditions are satisfied can researchers rely on the model to perform as expected and use it as a foundation to build our knowledge of the condition under study. However, a potential pitfall relates to the phenotypic differences between strains and substrains, and insufficient attention to this can lead to discrepancies. The substrains investigated here genetically diverged in the 1950s when separate breeding colonies were maintained in the Jacks Laboratory and NIH labs, hence the J and N strains and have been isolated for more than 200 generations.^23^ Despite their common origin, several studies have emphasized the numerous genomic changes including structural variations, indels, and single-nucleotide polymorphisms that distinguish the two strains,^23^ resulting in a large number of identified phenotypic differences^24^ including fundamental differences in cardiac physiology. C57BL/6N are often used for the development of HF models and demonstrate changes in connective tissue markers Nppa, and Myh7, and relatively more LV fibrosis following 14 days of angiotensin II infusion.^25^ Additionally, Zi *et al*.^26^ determined that C57BL/6N mice developed eccentric hypertrophy with cardiac deterioration depending on age and time course, whereas C57BL/6J mice developed variable cardiac phenotypes, when both strains underwent the same exposure to transverse aortic constriction.

In 2019, Schiattarella *et al.*^4^ reported a new murine model for HFpEF. Using the remarkably simple approach of induced obesity combined with high SBP, they were able to produce a range of signs consistent with HFpEF in a matter of weeks in N-substrain mice. Subsequently, Pepin *et al*.^11^ discussed the repeatability of this approach and the difficultly of reproducing this phenotype in other mouse strains. They showed that resistance to the induction of HFpEF segregated with a mutated form of the NNT gene that was harboured by the J strain. However, several phenotypic characteristics, including many of them relevant to HFpEF, such as energy metabolism, glucose tolerance, body weight, and response to cardiac insult could not be explained by alterations in the NNT gene alone,^17,27^ though we do agree the cardiac manifestations of HFpEF in J-strain mice are protected by the NNT mutation.

Many genetically modified mouse models are generated in C57BL/6J strains and as such they occupy a prominent place in model-based research. It was important, therefore, to investigate if it was possible to induce HFpEF in the resistant J strain. In this report, we demonstrate that utilizing HFD with a ramping dose of L-NAME to 1.75 g/L for 13 weeks sufficient to induce the essential cardiac features of human HFpEF in C57BL/6J mice, suggesting that a prolonged or intensified hypertensive stimulus may be necessary in the J substrain.

The literature contains mixed reports as a number of groups^8,9,12^ have successfully utilized J-strain mice to induce HFpEF at low doses of L-NAME and relatively short treatment periods of 5–8 weeks. These results are in stark contrast to the results reported here and elsewhere (Pepin *et al.*, 2025.^11^)

An additional complexity surrounding HFpEF murine models is incorporating the ageing component, a prominent risk factor in the human HFpEF patient that cannot be overlooked. As nitric oxide synthase activity, the target of L-NAME-induced hypertension, decreases with age,^28^ the L-NAME model is less effective in the aged rodent.

Recognizing these limitations, Deng *et al.*^5^ developed a ‘3-hit’ model, incorporating ageing as an additional factor to account for the increased risk of HFpEF in older populations. In this approach, 3-month-old mice are fed an HFD for 13 months to induce metabolic stress, and in the final month, DOCP is administered intraperitoneally to induce hypertension and systemic inflammation. This method has been regarded as the gold standard for HFpEF models, as it successfully integrates metabolic stress, hypertension, and aging—three key drivers of HFpEF, making it more representative of the human condition.^6,7^

DOCP, a long-acting synthetic mineralocorticoid, mimics the effects of aldosterone by binding to mineralocorticoid receptors in the kidney’s distal tubules, promoting sodium retention and potassium excretion. This increase in sodium retention leads to an expansion of extracellular fluid volume, which can elevate blood pressure. However, under normal physiological conditions, the body activates compensatory mechanisms, particularly the renin–angiotensin–aldosterone system (RAAS), to restore sodium balance and limit further increases in blood pressure.^29,30^

While exogenous DOCP administration leads to an initial retention of sodium, this effect is often transient. Over time, the phenomenon of ‘aldosterone escape’ occurs, wherein the kidneys adjust sodium excretion to match intake despite the continued presence of aldosterone.^31^ This adjustment helps prevent further fluid retention, although blood pressure may remain elevated. Thus, sodium retention alone may not be sufficient to sustain prolonged hypertension.

To overcome these compensatory mechanisms, our model combines DOCP with additional salt supplementation. While DOCP promotes sodium retention, the supplemental sodium load ensures that the kidneys are persistently challenged, preventing compensatory RAAS-driven sodium excretion and sustaining a positive sodium balance, leading to prolonged hypertension. By employing this strategy, we prevented aldosterone escape, sustained hypertension, and promoted fluid retention. Therefore, a ‘4-hit’ HFpEF model with 1% NaCl in drinking water as the fourth stressor is required to reproducibly induce HFpEF. Crucially, this model worked in both C57BL/6N and C57BL/6J strains, and in both male and female mice. Furthermore, it was observed that mice on the ‘4-hit’ HFpEF model could tolerate a second bolus of DOCP 8 weeks after the initial dose, when blood pressure started to decrease, with no adverse effects or sensitization responses. This was a crucial development, as it ensures the full HFpEF phenotype can be maintained for a meaningful period of time, allowing for the testing of therapeutic options.

When working with mouse models, animal welfare considerations should always be at the forefront, and an important modification to the 4-hit model was demonstrating effectiveness in a shorter timeframe. Studies have shown very long-term HFD exposure to mice can result in detrimental gut issues, dental abnormalities, and exacerbation of genetic predisposition to dermatitis.^32–34^ Furthermore, the desired metabolic effects of the HFD, such as weight gain and disturbance of glucose metabolism, are observed in as short as 7 weeks on the 2-hit L-NAME protocol, suggesting 13 months is unnecessary and expensive.

We also confirmed the same phenotype was generated when C57BL/6N mice were aged on healthy brown chow diet for 5 months, then swapped to HFD for 6 months (Supplementary Figures S2D–P). This resulted in the same age at sacrifice as those on HFD for 13 months, which means less unwanted side effects and suffering for mice, but no phenotypic compromise.

Taken together, our results confirm and extend prior findings. Our observations in the J-substrain mice confirm that a hypertensive stimulus via L-NAME can produce HFpEF features when dose or duration of exposure is increased. Additionally, our 4-hit model builds upon the established 3-hit design by incorporating NaCl supplementation to sustain hypertension, avoid aldosterone escape, and maintain the HFpEF phenotype.

## Conclusions

In this paper, we investigated the development of the 2-hit model of HFpEF in both C57BL/6N and C57BL/6J substrains. We demonstrated that it is possible to induce HFpEF in J-strain mice by increasing the dose or duration of exposure of L-NAME. This opens genetically engineered mouse models in both substrains for mechanistic and causal interrogation.

Furthermore, we provided a novel, robust, reproducible 4-hit ageing HFpEF model, which allows for greater clinical translation of results. We demonstrated this model to work in C57BL/6J and 6N male and female mice, and offered refinements to the protocol which still produce a strong phenotype whilst satisfying animal welfare needs. We also demonstrated that the 4-hit model can sustain the HFpEF phenotype for up to 12 weeks, enabling therapeutic options to be tested in this setting.

## Limitations

Although the models herein are the most commonly used and critically appraised murine HFpEF models, they are not the only HFpEF models in use, and other aspects of HFpEF such as chronic pulmonary and renal disease are not incorporated. Furthermore, while this study provides a refined 2-hit L-NAME protocol for the C57BL/6J substrain, these optimization experiments were conducted in male mice. The efficacy of the ramp-up protocol in female J-strain mice remains to be determined. Moreover, the study did not assess glucose tolerance in the refined C57BL/6J 2-hit model, a phenotype is well-established in the C57BL/6N strain. The E/A data are unavailable for the J1 mice (*Figure 2*) due to technical unavailability of the instrument. Furthermore, we recognize that VO_2_ max is the gold standard for comparing exercise capacity between groups and acknowledge the absence of this in the modified 2-hit groups.

We employed E/A ratio to assess diastolic dysfunction in the 2-hit model. While these parameters are well recognized and widely used surrogates for diastolic dysfunction, they have limitations in mouse models due to rapid heart rates. Parameters such as left atrial area and reverse longitudinal strain rate may offer more robust insights into diastolic dysfunction; however, they were not included in the current study due to technical constraints. Future studies need to also incorporate PV loop experiments as an additional approach to validate and strengthen the echocardiographic findings. Additionally, obtaining reliable interventricular septal thickness measurements using echocardiography was technically not feasible.

Some datasets had a sample size of *n* = 4 (forced exercise testing and cardiomyocyte cross-sectional area; *Figures 1I, 2F,* and *2O*) mainly due to practical and ethical considerations. Firstly, not all animals run on the exercise wheel, therefore running distance can only be assessed for limited number of animals. Secondly, the model required iterative optimization, and the use of a relatively high dose of L-NAME (1.5–1.75 mg/L) necessitated an initial small-cohort safety and feasibility pilot conducted in compliance with institutional and British Heart Foundation animal welfare guidelines in the UK. Consequently, early experiments were limited in sample size and statistical power but were essential to ensure animal safety and refine the experimental design prior to larger studies.

## Supplementary Material

xvag072_Supplementary_Data

## Data Availability

No data were generated or analysed for this manuscript.

## Pre-registered Clinical Trial Number

None supplied.
