# Supplementary material for: Optimized murine HFpEF models for translational preclinical studies
Source: ESC Heart Fail. 2026 Mar 11;13(2):xvag072. doi: 10.1093/eschf/xvag072 (PMC13036831; doi:10.1093/eschf/xvag072)
Supplement: xvag072_Supplementary_Data [file xvag072_supplementary_data.zip › Optimised murine HFpEF models_Supplemental Figures Final .docx]

**Supplemental Figure 1**

**
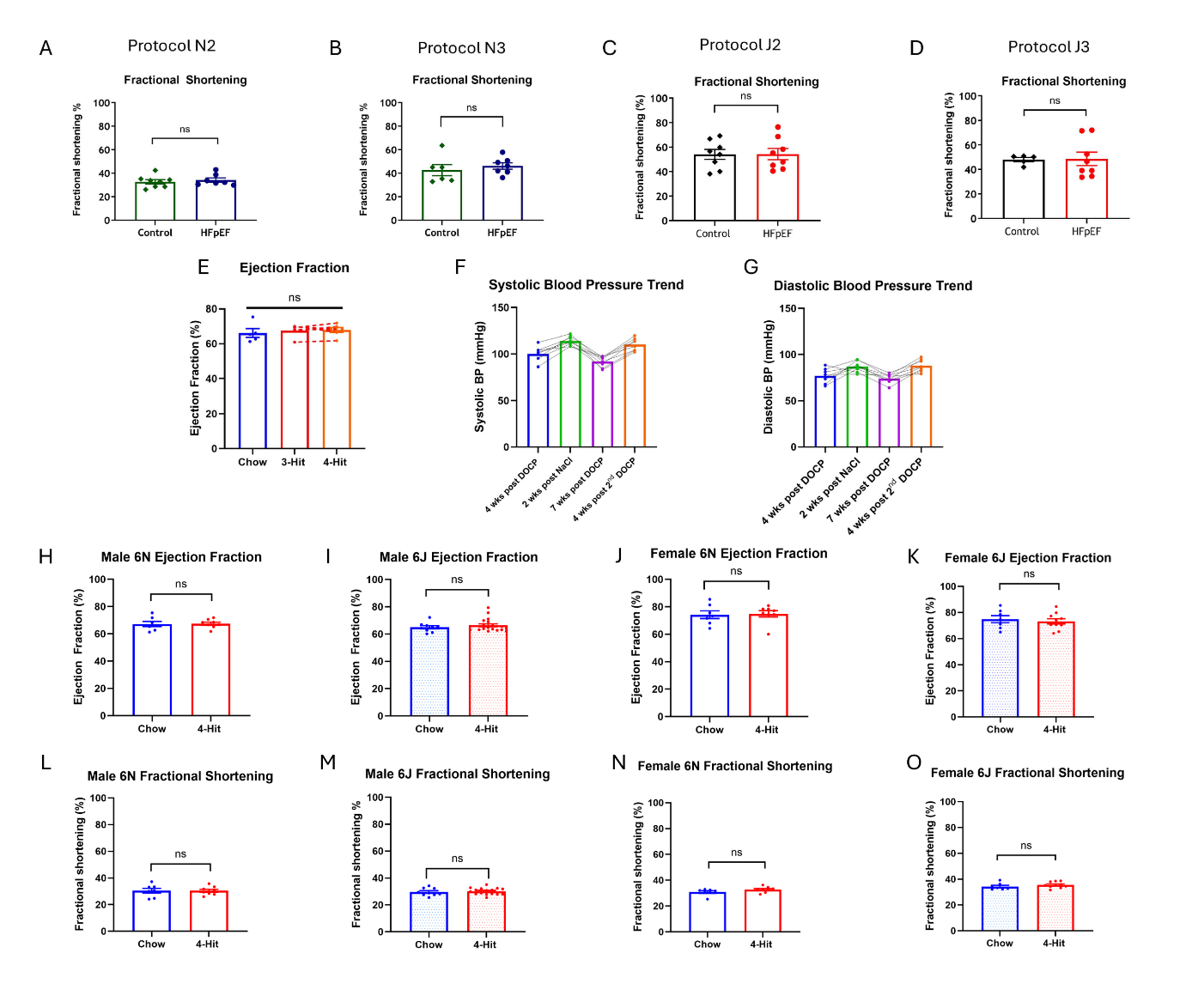
**

**Supplemental Figure. 1. Maintenance of ejection fraction and blood pressure. A**. Fractional shortening maintained in male mice at conclusion of protocol N2 (*n*=6 for control, *n*=7 for treated). **B**. Fractional shortening maintained in male mice at conclusion of protocol N3 (*n*=6 for control, *n*=7 for treated). **C**. Fractional shortening maintained in male mice at conclusion of protocol J2 (*n*=8 for control, *n*=8 for treated). **D**. Fractional shortening maintained in male mice at conclusion of J3 protocol (*n*=5 for control, *n*=8 for treated). **E**. Preserved ejection fraction (EF) of male 6N mice before and after the addition of NaCl drinking water (*n*=6 for chow, *n*=7 for 3-Hit, *n*=7 for 4-Hit) **F**. Trend of systolic blood pressure in 6N mice between DOCP dosing and addition of NaCl (*n*=7). **G**. Trend of diastolic blood pressure in 6N mice between DOCP dosing and addition of NaCl (*n*=7). **H.** EF of male 6N mice at conclusion of 4-Hit protocol (*n*=7 for chow, *n*=8 for 4-Hit). **I**. EF of male 6J mice at conclusion of 4-Hit protocol (*n*=9 for chow, *n*=19 for 4-Hit). **J**. EF of female 6N mice at conclusion of 4-Hit protocol (*n*=7 for chow, *n*=8 for 4-Hit). **K**. EF of female 6J mice at conclusion of 4-Hit protocol (*n*=7 for chow, *n*=10 for 4-Hit). **L.** Fractional shortening (FS) of male 6N mice at conclusion of 4-Hit protocol (*n*=7 for chow, n=8 for 4-Hit). **M**. FS of male 6J mice at conclusion of 4-Hit protocol (*n*=9 for chow, *n*=19 for 4-Hit). **N**. FS of female 6N mice at conclusion of 4-Hit protocol (*n*=7 for chow, *n*=8 for 4-Hit). **O**. FS of female 6J mice at conclusion of 4-Hit protocol (*n*=7 for chow, *n*=10 for 4-Hit). NS determined as P>0.05 by Mann-Whitney test. Graphs display ± SEM.

**Supplemental Figure 2
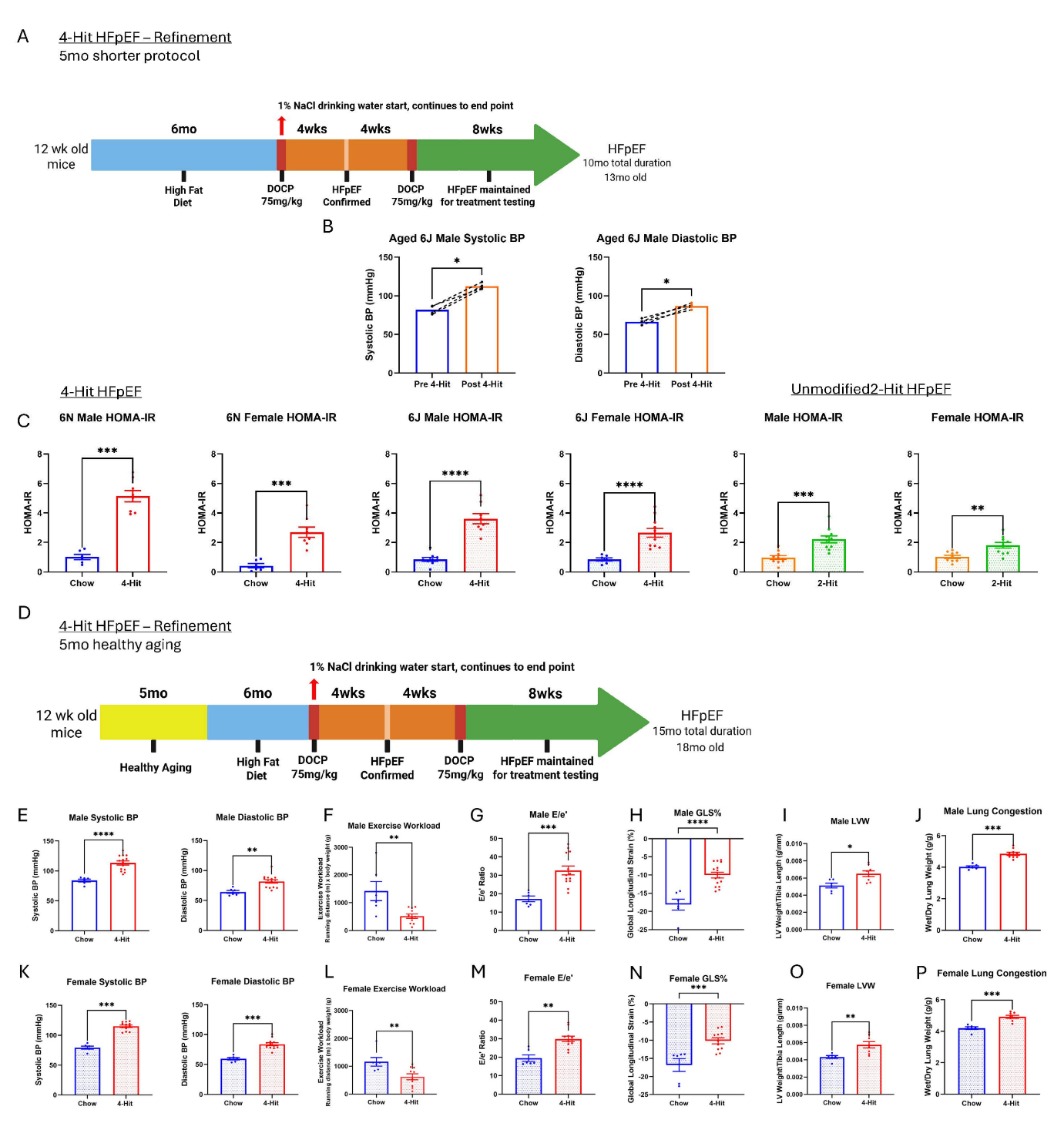
**

**Supplemental Figure. 2. Refinements to the 4-Hit model for improved animal welfare. A.** Schematic of a refinement option for 4-Hit model with high fat diet (HFD) loading phase shortened by 5 months, for overall duration of 11 months. **B**. Systolic and diastolic blood pressure of male 6J mice after full duration of 13 months aging, DOCP injection and NaCl drinking water (n=4). **C**. HOMA-IR from male and female 6N and 6J mice on the 4-Hit protocol (complementary data to figure 4) and male and female 6J mice on the unmodified 2-Hit protocol (complementary data to figure 5). **D.** A schematic for refinement option for 4-Hit model including 5 months of healthy aging then 6 months high fat diet, for 15 months total duration. Panels D-O depict measurements from male and female 6J mice after completion of the refined 4-Hit model outlined in panel C. **E**. Systolic and diastolic blood pressure of male mice. **F**. Exercise workload of male mice. **G**. Ratio of peak velocity of mitral blood flow at early filling to peak early diastolic mitral annulus velocity (E/e’). **H**. Global longitudinal strain (GLS) of the left ventricle. **I**. Left ventricular weight to tibia length as an indicator of left ventricular hypertrophy. **J**. Wet lung weight to tibia length as an indicator of lung congestion. For D-I, *n*=6 for chow, *n*=14 for 4-Hit. **K**. Systolic and diastolic blood pressure of female mice. **L**. Running distance of female mice. **M**. Ratio of peak velocity of mitral blood flow at early filling to peak early diastolic mitral annulus velocity (E/e’). **N**. Global longitudinal strain (GLS) of the left ventricle. **O**. Left ventricular weight to tibia length as an indicator of left ventricular hypertrophy. **P**. Wet lung weight to tibia length as an indicator of lung congestion. For J-O, *n*=6 for chow, *n*=10 for 4-Hit. *P<0.05, **P<0.01, ***P<0.001, ****P<0.0001 by Mann-Whitney test. Graphs display ± SEM.
