# Supplementary material for: Optimized murine HFpEF models for translational preclinical studies
Source: ESC Heart Fail. 2026 Mar 11;13(2):xvag072. doi: 10.1093/eschf/xvag072 (PMC13036831; doi:10.1093/eschf/xvag072)
Supplement: xvag072_Supplementary_Data [file xvag072_supplementary_data.zip › Supplemental Data Table 1.docx]

| ***Echocardiography*** |  | |  | |
| --- | --- | --- | --- | --- |
| **2-Hit Protocols** | |  | |  |
| **Protocol N1** | | **Control (n=12)** | | **HFpEF (n=12)** |
| LVID,d (mm) | | 3.679 ± 0.213 | | 3.429 ± 0.252 |
| LVID, s (mm) | | 1.659 ± 0.169 | | 1.305 ± 0.170 |
| LVFS (%) | | 34.220 ± 2.302 | | 35.455 ± 3.075 |
| Peak mitral E velocity (mm/sec) ** | | 430.777 ± 16.344 | | 491.291 ± 8.606 |
| Peak mitral A velocity (mm/sec) **** | | 309.418 ± 12.809 | | 229.133 ± 10.318 |
| Mitral E/A *** | | 1.307 ± 0.099 | | 2.241 ± 0.178 |
|  | |  | |  |
| **Protocol N2** | | **Control (n=8)** | | **HFpEF (n=8)** |
| LVID,d (mm) | | 4.510 ± 0.083 | | 4.245 ± 0.095 |
| LVID, s (mm) | | 3.093 ± 0.071 | | 2.987 ± 0.106 |
| LVFS (%) | | 32.711 ± 1.805 | | 34.202 ± 1.840 |
| Peak mitral E velocity (mm/sec) **** | | 444 ± 13.233 | | 564.340 ± 15.246 |
| Peak mitral A velocity (mm/sec) **** | | 281.280 ± 6.078 | | 200.524 ± 6.595 |
| Mitral E/A **** | | 1.616 ± 0.053 | | 2.854 ± 0.115 |
|  | |  | |  |
| **Protocol N3** | | **Chow (n=6)** | | **HFpEF (n=7)** |
| LVID,d (mm) | | 4.519 ± 0.260 | | 4.249 ± 0.134 |
| LVID, s (mm) | | 2.646 ± 0.313 | | 2.397 ± 0.169 |
| LVFS (%) | | 42.564± 4.754 | | 46.120 ± 2.705 |
| Peak mitral E velocity (mm/sec) **** | | 338.989 ± 18.711 | | 481.372 ± 8.123 |
| Peak mitral A velocity (mm/sec) ** | | 292.285 ± 19.435 | | 222.784 ± 5.844 |
| Mitral E/A *** | | 1.218 ± 0.136 | | 2.183 ± 0.078 |
|  | |  | |  |
| **Protocol J2** | | **Chow (n=8)** | | **HFpEF (n=8)** |
| LVID,d (mm) | | 3.947 ± 0.141 | | 3.602 ± 0.211 |
| LVID, s (mm) | | 1.790 ± 0.134 | | 1.613 ± 0.164 |
| LVFS (%) | | 54.082 ± 4.047 | | 54.292 ± 4.573 |
| Peak mitral E velocity (mm/sec) | | 384.586 ± 16.432 | | 394.740 ± 14.750 |
| Peak mitral A velocity (mm/sec) * | | 293.845 ± 9.589 | | 246.137 ± 13.178 |
| Mitral E/A | | 1.342 ± 0.124 | | 1.804 ± 0.327 |
|  | |  | |  |
| **Protocol J3** | | **Chow (n=8)** | | **HFpEF (n=5)** |
| LVID,d (mm) | | 3.518 ± 0.503 | | 3.247 ± 0.197 |
| LVID, s (mm) | | 1.849 ± 0.296 | | 1.698 ± 0.223 |
| LVFS (%) | | 47.991 ± 1.722 | | 48.599 ± 5.503 |
| Peak mitral E velocity (mm/sec) *** | | 254.268 ± 10.226 | | 366.607 ± 14.388 |
| Peak mitral A velocity (mm/sec) | | 169.881 ± 7.560 | | 185.449 ± 9.592 |
| Mitral E/A * | | 1.538 ± 0.112 | | 1.991 ± 0.101 |
|  | |  | |  |
| **4-Hit Protocols** | | | | |
| **6N Male** | | **Chow (n=7)** | | **4-Hit (n=8)** |
| LVID,d (mm) | | 4.137 ± 0.149 | | 3.883 ± 0.191 |
| LVID, s (mm) | | 2.807 ± 0.142 | | 2.694 ± 0.174 |
| LVFS (%) | | 29.064 ± 1.981 | | 33.419 ± 0.870 |
| LVEF (%) | | 67.267 ± 1.880 | | 67.478 ± 1.116 |
| Peak mitral E velocity (mm/sec) ** | | 444.505 ± 9.735 | | 511.636 ± 11.542 |
| Peak mitral E’ velocity (mm/sec) ** | | 29.599 ± 1.117 | | 15.267 ± 0.935 |
| Mitral E/E’ *** | | 17.341 ± 1.547 | | 34.778 ± 1.331 |
| Global longitudinal strain (%) *** | | -16.526 ± 0.670 | | 9.658 ± 0.259 |
|  | |  | |  |
| **6N Female** | | **Chow (n=7)** | | **4-Hit (n=8)** |
| LVID,d (mm) | | 3.492 ± 0.118 | | 3.890 ± 0.136 |
| LVID, s (mm) | | 2.359 ± 0.094 | | 2.611 ± 0.135 |
| LVFS (%) | | 30.583 ± 1.110 | | 32.121± 1.316 |
| LVEF (%) | | 74.234 ± 2.771 | | 74.881 ± 2.265 |
| Peak mitral E velocity (mm/sec) ** | | 373.590 ± 15.00 | | 465.537 ± 12.857 |
| Peak mitral E’ velocity (mm/sec) ** | | 23.519 ±1.392 | | 15.981 ± 0.985 |
| Mitral E/E’ *** | | 15.878 ± 0.503 | | 30.528 ± 1.243 |
| Global longitudinal strain (%) *** | | -20.490 ± 0.954 | | -10.377 ± 0.710 |
|  | |  | |  |
| **6J Male** | | **Chow (n=9)** | | **4-Hit (n=8)** |
| LVID,d (mm) | | 4.184 ± 0.150 | | 4.226 ± 0.178 |
| LVID, s (mm) | | 2.957 ± 0.109 | | 3.040 ± 0.156 |
| LVFS (%) | | 30.165 ± 1.007 | | 31.128 ± 0.484 |
| LVEF (%) | | 65.030 ± 1.191 | | 66.928 ± 1.725 |
| Peak mitral E velocity (mm/sec) * | | 470.716 ± 9.270 | | 491.937 ± 7.518 |
| Peak mitral E’ velocity (mm/sec) **** | | 26.180 ± 1.643 | | 13.127 ± 1.034 |
| Mitral E/E’ **** | | 19.539 ± 0.662 | | 40.706 ± 1.503 |
| Global longitudinal strain (%) **** | | -16.080 ± 0.643 | | -6.914 ± 0.258 |
|  | |  | |  |
| **6J Female** | | **Chow (n=7)** | | **4-Hit (n=10)** |
| LVID,d (mm) | | 3.851 ± 0.079 | | 4.043 ± 0.096 |
| LVID, s (mm) | | 2.673 ± 0.106 | | 2.799 ± 0.073 |
| LVFS (%) | | 35.566 ± 1.148 | | 35.529 ± 0.760 |
| LVEF (%) | | 74.893 ± 2.767 | | 73.216 ± 2.001 |
| Peak mitral E velocity (mm/sec) ** | | 454.462 ± 8.5200 | | 556.895 ± 12.508 |
| Peak mitral E’ velocity (mm/sec) *** | | 24.640 ± 0.940 | | 16.425 ± 1.154 |
| Mitral E/E’ **** | | 15.903 ± 0.770 | | 35.534 ± 0.857 |
| Global longitudinal strain (%) **** | | -19.108 ± 0.569 | | -10.631 ± 0.398 |

Values are presented as mean ± SEM. Mann-Whitney test was used to detect significance.  *P<0.05, **P<0.01, ***P<0.001, ****P<0.0001. Echocardiography parameters were acquired under isoflurane with heart rates maintained between 420 and 520bpm. LVIDd, left ventricular internal diastolic diameter; LVIDs, left ventricular internal systolic diameter; LVFS, left ventricular fractional shortening; LVEF, left ventricular ejection fraction; E, peak doppler blood flow velocity across the mitral valve during early diastole; A, peak doppler blood flow velocity across the mitral valve during late diastole; E’, peak tissue doppler at mitral annulus of myocardial relaxation velocity during early diastole.
